# Deep Vein Thrombosis Secondary to Brucellosis: An Uncommon Infectious Cause of Venous Thrombosis

**DOI:** 10.1155/crdi/5004349

**Published:** 2026-08-11

**Authors:** Ghenwa El Dakdouki, Doha Daoud, Roa Osman, Walid Moety, Rim Youssef, Sara Farhat

**Affiliations:** ^1^ Internal Medicine Department, Infectious Diseases Division, Hammoud Hospital University Medical Center, Sidon, Lebanon, hammoudhospital.com; ^2^ Faculty of Medicine, Beirut Arab University, Beirut, Lebanon, bau.edu.lb; ^3^ Faculty of Medicine, Lebanese University, Beirut, Lebanon, ul.edu.lb

**Keywords:** brucellosis, case report, deep vein thrombosis, hypercoagulability, zoonosis

## Abstract

**Introduction:**

Brucella is a Gram‐negative coccobacillus responsible for a zoonotic infection that is endemic in the Mediterranean region, including Lebanon. Although it commonly presents with systemic and musculoskeletal manifestations, vascular complications such as deep vein thrombosis (DVT) are rarely reported. This case highlights an uncommon thrombotic complication of brucellosis and contributes to the limited literature on brucellosis‐associated DVT, particularly in endemic regions.

**Case Presentation:**

We report the case of a previously healthy 33‐year‐old male from Bekaa, Lebanon, who presented with prolonged fever and bilateral lower limb pain and swelling. Doppler ultrasound confirmed bilateral DVT. Extensive investigations for thrombophilia, malignancy, and autoimmune diseases were unremarkable. Brucella indirect serology was strongly positive with a titer of 1:1280, confirming active infection. The patient was treated with streptomycin, rifampicin, doxycycline, and anticoagulation therapy, resulting in marked clinical improvement and sustained recovery during a 3‐year follow‐up period.

**Conclusion:**

This case report highlights the importance of considering brucellosis as a cause of unexplained DVT, mainly in endemic regions, especially in the absence of a family history of hypercoagulable disorders.

## 1. Introduction

Human brucellosis is a zoonotic disease caused by facultative intracellular, Gram‐negative coccobacilli of the genus *Brucella* [1]. It has a worldwide distribution and is particularly endemic in the Mediterranean region, including Lebanon [2]. Transmission occurs mainly through ingestion of contaminated dairy products or through direct contact with infected animals [1]. It can present with a wide range of systemic clinical manifestations, ranging from mild, nonspecific symptoms to severe systemic involvement [3, 4]. Deep vein thrombosis (DVT) is a rare manifestation of this disease [5]. Brucellosis‐associated DVT is a rare complication, with limited cases reported worldwide. To our knowledge, this is the first reported case of bilateral DVT secondary to brucellosis in Lebanon. This case highlights an uncommon thrombotic manifestation of brucellosis and emphasizes the importance of considering infectious causes in patients with unexplained venous thrombosis, particularly in endemic regions. Herein, we report the case of a previously healthy patient who presented with bilateral DVT secondary to brucellosis.

## 2. Case Presentation

A 33‐year‐old healthy male from Bekaa, Lebanon, presented with a high‐grade fever of one month duration, followed by severe bilateral lower limb pain and swelling.

On presentation, the patient appeared ill with a temperature of 40°C. He was tachycardic (HR: 110/min) and normotensive (BP: 110/70 mmHg). Physical examination revealed severe right thigh swelling and tenderness with localized erythema and warmth.

History taking revealed that our patient was from the Bekaa region, an area where brucellosis is endemic. Patient reported exposure to locally produced dairy products, the type of processing, whether pasteurized or not, could not be confirmed.

Initial laboratory investigations were unremarkable except for elevated infection marker with a neutrophil predominance suggestive of bacterial infection. Results are summarized in Table 1. The echo Doppler of the lower extremities showed bilateral DVT, more prominent in the right thigh.

**TABLE 1:** Summary of laboratory investigations.

Lab tests		Result	Reference range
WBC		8890/mm^3^	4000–11,000/mm^3^
	Neutrophils	70%	40%–70%
	Lymphocytes	18%	20%–40%
	Monocytes	6.70%	2%–8%
Hemoglobin		14.5 g/dL	13.5–17.5 g/dL
Platelet count		213,000/mm^3^	150,000–450,000/mm^3^
BUN		11 mg/dL	7–20 mg/dL
Creatinine		0.6 mg/dL	0.6–1.3 mg/dL
ALT		9 IU/L	7–56 IU/L
CRP		196 mg/L	< 5 mg/L
Protein C		103%	70%–140%
Protein S		96.60%	63%–135%
Antithrombin III		99.40%	83%–128%
Homocysteine		15.9 μmol/L	5.46–16.2
ANA		Negative	
Anti‐dsDNA		< 10 IU/mL	< 30 IU/mL
RF		18.9 IU/mL	< 14.1
Lupus anticoagulant		Negative	Negative
Antiphospholipid antibodies		Negative	Negative
Anticardiolipin IgG		< 10 GPL U/mL	< 20 GPL U/mL
Anticardiolipin IgM		< 10 MPL U/mL	< 20 MPL U/mL
TSH		2.1 µIU/mL	0.4–4.0 µIU/mL

A full workup was performed to rule out underlying causes of DVT, such as malignancy or coagulopathy. Computed Tomography (CT) Scan of the chest, abdomen, and pelvis with contrast revealed moderate homogeneous splenomegaly without other abnormalities. Magnetic resonance imaging of the right thigh demonstrated diffuse edematous thickening involving mainly the rectus and quadriceps muscles with ill‐defined venous enhancement. These findings were most consistent with myositis and cellulitis, without evidence of abscess formation or osteomyelitis. Bone marrow aspirate and biopsy showed hypercellularity with adequate megakaryocytes, normal erythroid series, and active myeloid series.

In addition, a complete coagulation and autoimmune workup was within normal limits. Results are summarized in Table 1.

Given the high index of clinical suspicion, Brucella titers were obtained. The Brucella standard agglutination test (SAT) was negative (titer < 1:80), whereas the Brucella Coombs test (indirect antiglobulin test), detecting anti‐Brucella blocking antibodies, revealed a high titer of 1:1280, supporting the diagnosis of brucellosis. Blood cultures were also obtained and yielded negative results.

Our patient presented with a 1‐month history of high‐grade fever followed by bilateral lower limb swelling and pain. He was hospitalized for further evaluation, and a diagnostic workup revealed bilateral DVT associated with brucellosis. After diagnosis, he received streptomycin 1‐g IM daily for 2 weeks, rifampicin 600‐mg orally daily for 3 months, and doxycycline 100‐mg PO twice daily for 3 months, in addition to therapeutic doses of low molecular weight heparin for 3 months, followed by oral anticoagulants for another 3 months. The timeline of clinical events is summarized in Table 2.

**TABLE 2:** Timeline of clinical events.

1 month before admission	High‐grade fever
At presentation	High‐grade fever, tachycardia, and bilateral lower limb swelling and pain
Hospital course	Laboratory investigations, echo Doppler, CT chest abdomen pelvis, MRI, bone marrow biopsies, thrombophilia workup. Brucella was diagnosed
After diagnosis	Antibiotic course and anticoagulants
Follow‐up	The patient reported significant symptomatic improvement following treatment and was able to return to normal daily activities without recurrence of symptoms during follow‐up.

The patient showed marked clinical improvement with complete resolution of symptoms. He completed the prescribed treatment regimen without reported adverse effects. During a 3‐year follow‐up period, the patient remained clinically stable without recurrence of thrombotic events or brucellosis‐related symptoms.

## 3. Discussion

Brucellosis, also referred to as “Malta fever” and “Mediterranean fever,” was first described in 1887 by Sir David Bruce [6]. It is transmitted via contaminated food (unpasteurized dairy and raw meat) or direct contact with secretions from infected animals [1, 7]. Although many patients remain asymptomatic, symptomatic disease typically presents with nonspecific manifestations such as arthralgia, fever, night sweats, weight loss, nausea, vomiting, headache, back pain, and myalgia [1, 6, 7]. In addition, it might cause several complications, including endocarditis [8, 9], epididymoorchitis, spondylodiscitis, and meningitis [10].

Although vascular complications are rare, we report a rare case of DVT secondary to Brucella infection. In our patient, the hypercoagulability and autoimmune workup, except for a mildly elevated rheumatoid factor (RF) (RA latex), were negative, while the Brucella Coombs test, which detects anti‐Brucella blocking antibodies, was positive, confirming active infection. Therefore, we hypothesize that this venous thromboembolism is directly related to the infection itself or its toxins, as other risk factors are absent, such as immobilization, malignancy, recent surgical intervention, or trauma.

Because bilateral DVT presented in a young, previously healthy individual, a broad differential diagnosis was considered. Potential etiologies included inherited thrombophilias, antiphospholipid syndrome, occult malignancy, venous compression, and systemic inflammatory or infectious disorders. Therefore, a thrombophilia workup, an autoimmune workup, and imaging studies to rule out occult malignancy were performed; however, they failed to identify a clear underlying cause. Differential diagnoses for prolonged fever, splenomegaly, and musculoskeletal manifestations, including other infectious or inflammatory causes were also considered; however, our patient’s epidemiological history and labs supported the diagnosis of brucellosis.

The diagnosis was particularly challenging because the Brucella SAT was negative, despite a positive Brucella Coombs test. This discrepancy can be explained by the difference in antibody detection between these two tests. The SAT primarily detects agglutinating antibodies, whereas the Coombs test detects incomplete or blocking nonagglutinating antibodies that may not produce visible agglutination. Previous studies show that Coombs may provide higher diagnostic sensitivity, and SAT titers may be low or negative in early, subacute, chronic, or partially treated brucellosis, while Coombs test titers remain positive and can still be detected. This serological pattern highlights the importance of using complementary tests when clinical suspicion remains high despite a negative SAT result [11].

Another diagnostic challenge in our patient was the mildly elevated RF level. RF is not specific for rheumatoid arthritis and may also be elevated in various infectious and inflammatory conditions [12, 13]. This phenomenon is thought to result from infection‐induced polyclonal B‐cell activation, which stimulates the production of multiple antibodies, including RF(12). Therefore, accurate diagnosis requires careful correlation of the patient’s clinical presentation, exposure history, and laboratory findings to distinguish brucellosis from other conditions with similar serological profiles.

Our review of the literature identified only 12 reported cases of brucellosis‐associated DVT (Table 3), underscoring the exceptional rarity of this complication. In addition, other vascular sites affected by brucellosis have also been described, including the inferior vena cava (IVC) [22], portal vein [20], cerebral sinuses [21], and retinal vein thrombosis [9] (Table 3).

**TABLE 3:** Reported cases of Brucella and DVT in the literature.

Author	Year	Country	Gender	Age	Vein thrombosis	Serology	Antibiotic therapy (days)	Anticoagulant therapy
Marfil Rivera et al. [14]	1986	Mexico	F	18	Lower Limb	NM	Tetracycline	—
Odeh et al. [5]	2000	Israel	M	52	Right leg	1/160	R + D	Warfarin for 3 months
Memish et al. [15]	2001	Saudi Arabia	M	41	Right leg	1/1280	R + D	Heparin for 6 weeks
Gul et al. [16]	2008	Turkey	M	21	Right leg (femoral vein)	1/400	R + D (42 days)	Warfarin for 5 months
Tolaj et al. [9]	2014	Kosovo	M	37	Left leg	1/640	Streptomycin + D (2 w), D + R (6 m)	Warfarin for 5 months
Kocak et al. [4]	2012	Turkey	M	26	Right leg	1/320	R + D	Enoxaparin 60 mg sc, then heparin 20000 u sc, and then warfarin 5 mg po
Koubaa et al. [8]	2012	Tunisia	M	45	Left leg (femoral vein)	1/640	R + D (90 days)	LMWH for 3 months
Davoudi et al. [11]	2014	Iran	M	15	Left leg	1/1280	R + D + Amikacin (NM)	LMWH for 1 week and then warfarin for 6 months
Salihi et al. [17]	2016	Turkey	M	26	Right leg	1/160	R + D (NM)	Heparin 20000 u iv and then warfarin 5 mg po
Ghafouri et al. [18]	2021	Iran	M	62	Right leg	1/1280	R + TS + Azithromycin (NM)	Rivaroxaban 15 mg twice a day for 3 w, then 20 mg once a day + enoxaparin 60 u sc twice a day, and then warfarin for 6 months
Singh gopal et al. [19]	2024	India	M	44	Bilateral legs	1/320	R + d (6M)	LMWH for 5 days and then warfarin for 3 months
Romem et al. [7]	1973	Israel	NM	NM	Central retinal vein	NM	NM	—
Gregori et al. [20]	1990	Spain	NM	NM	Portal vein	NM	NM	Heparin and then surgery
Zaidan et al. [21]	1999	Saudi Arabia	F	23	Cerebral vein (sagittal sinus)	1/320	R + D + TS	—
Rueggr et al. [22]	2017	Switzerland	F	23	Inferior vena cava	NM	NM	—
Present case	2015	Lebanon	M	33	Bilateral, more on right	1/1280	Streptomycin (2 w) + rifampicin + doxycycline (3 m)	Therapeutic doses of low molecular weight heparin for 3 months, followed by oral anticoagulants for another 3 months

*Note:* F, female; R, rifampicin; D, doxycycline; M, male; T, tetracycline.

Abbreviations: NM, not mentioned; TS, trimethoprim–sulfamethoxazole.

Given the rarity of this complication, understanding its pathogenesis is important. Although the exact mechanism is not fully understood, intracellular proliferation of the organism induces endothelial injury and activates inflammation [1, 5, 9]. Consequently, this promotes platelet adhesion and hypercoagulability, fulfilling components of Virchow’s triad that predispose to venous thrombosis [8, 16, 17].

To further elucidate this process, Brucella outer membrane lipoproteins directly activate platelets, which in turn stimulate endothelial cells via the ERK1/2 MAP kinase signaling pathway. This signaling cascade upregulates the expression of endothelial adhesion molecules, including ICAM‐1 and CD40, and increases the secretion of proinflammatory cytokines and chemokines, such as IL‐6, IL‐8, and CCL2 [23]. These mediators promote the recruitment and adhesion of neutrophils and monocytes to the vascular endothelium, amplifying the inflammatory response and contributing to endothelial dysfunction. Furthermore, proinflammatory cytokines, particularly TNF‐α and IL‐6, activate the coagulation cascade and further enhance the prothrombotic state, especially in the presence of additional risk factors.

Collectively, these mechanisms promote venous clot formation and ultimately lead to DVT.

Furthermore, other probable mechanisms have been discussed in the literature. During an ongoing infectious process, collections such as abscesses can compress nearby veins, causing stasis and subsequent clot formation. Moreover, inflammation of the vessel walls increases the likelihood of thrombosis [8, 9].

Recognizing this association is crucial as it guides the therapeutic approach. Accordingly, the treatment aim is to prevent the formation of clots while treating the infection itself with appropriate antibiotics (rifampicin and doxycycline). This approach is consistent with previously reported cases (Table 3).

## 4. Conclusion

In conclusion, brucellosis is an insidious organism and a potential cause of severe and even fatal complications, including DVT. Thus, in endemic areas, brucellosis should always be considered in the differential diagnosis of patients with unexplained venous thrombosis. Early diagnosis, prompt, and adequate management, with a proper follow‐up, are essential.

## 5. Strengths

This case has several strengths. To our knowledge, it represents the first reported case of Brucella‐associated bilateral DVT in Lebanon and only the second reported case worldwide. Reporting this rare complication from an endemic region is important because it emphasizes the need to consider brucellosis in the differential diagnosis of unexplained DVT, even when the SAT is negative and clinical suspicion remains high.

## 6. Limitations

One limitation of this report is that the original radiology reports were unavailable for review during manuscript preparation. However, the findings were documented in the patient’s medical records and were consistent with the clinical course of the patient.

Although an extensive thrombophilia workup was done, testing for factor V Leiden and prothrombin G20210A mutation was not available and this represents a limitation.

**Nomenclature**
DVT: Deep venous thrombosis
WBC: White blood cell count
BUN: Blood urea nitrogen
ALT: Alanine aminotransferase
CRP: C‐reactive protein
AN: Antinuclear antibodies
RF: Rheumatoid factor
Anti‐dsDNA: Anti‐double‐stranded DNA antibodies
IM: Intramuscular
SAT: Standard agglutination test

## Author Contributions

All authors contributed to the conception, clinical management, drafting, and revision of the manuscript.

## Funding

No funding was received for this study.

## Disclosure

All authors have approved the final version of the manuscript.

## Ethics Statement

Ethical approval was not required for this single case report according to institutional policy. Written informed consent was obtained from the patient.

## Consent

Written informed consent was obtained from the patient for publication of this case report and accompanying data.

## Conflicts of Interest

The authors declare no conflicts of interest.

## Patient Perspective

The patient reported significant symptomatic improvement following treatment and was able to return to normal daily activities without recurrence of symptoms during follow‐up.

## Supporting Information

Additional supporting information can be found online in the Supporting Information section.

## Supporting information


**Supporting Information** CARE Checklist of information to include when writing a case report.

## Data Availability

All clinical data supporting the findings of this case report are included within the article.
