# Supplementary material for: Deep Vein Thrombosis Secondary to Brucellosis: An Uncommon Infectious Cause of Venous Thrombosis
Source: Case Rep Infect Dis. 2026 Aug 11;2026:5004349. doi: 10.1155/crdi/5004349 (PMC13462663; doi:10.1155/crdi/5004349)
Supplement: Supplementary file 1 [file CRDI-2026-5004349-s001.pdf]

CARE
case report guidelines

CARE Checklist of information to include when writing a case report

(cc) BY-NC-ND

Topic	Item	Checklist item description	Reported on Line
**Title**	**1**	The diagnosis or intervention of primary focus followed by the words "case report"	lines 1-2
**Key Words**	**2**	5 k y words that identify diagnoses or interventions in this case report, including "case report"	line 28
**Abstract (no references)**	**3a**	Introduction: What is unique about this case and what does it add to the scientific literature?	lines 15-16-17
	**3b**	Main symptoms and/or important clinical findings	line 20
	**3c**	The main diagnoses, therapeutic interventions, and outcomes	line 21-24-25
	**3d**	Conclusion—What is the main "take-away" lesson(s) from this case?	line 27
**Introduction**	**4**	One or two paragraphs summarizing why this case is unique (**may include references**)	line 38->42
**Patient Information**	**5a**	De-identified patient specific information.	line 46
	**5b**	Primary concerns and symptoms of the patient.	line 46-47
	**5c**	Medical, family, and psycho-social history including relevant genetic information	No relevant history was reported.
	**5d**	Relevant past interventions with outcomes	No relevant previous interventions were reported.
**Clinical Findings**	**6**	Describe significant physical examination (PE) and important clinical findings.	lines 48-49-50
**Timeline**	**7**	Historical and current information from this episode of care organized as a timeline	lines 70->77
**Diagnostic Assessment**	**8a**	Diagnostic testing (such as PE, laboratory testing, imaging, surveys).	lines 49->64
	**8b**	Diagnostic challenges (such as access to testing, financial, or cultural)	none
	**8c**	Diagnosis (including other diagnoses considered)	lines 63-64
	**8d**	Prognosis (such as staging in oncology) where applicable	not included
**Therapeutic Intervention**	**9a**	Types of therapeutic intervention (such as pharmacologic, surgical, preventive, self-care)	lines73->76
	**9b**	Administration of therapeutic intervention (such as dosage, strength, duration)	lines 73->76
	**9c**	Changes in therapeutic intervention (with rationale)	none
**Follow-up and Outcomes**	**10a**	Clinician and patient-assessed outcomes (if available)	lines 77-80
	**10b**	Important follow-up diagnostic and other test results	line 79
	**10c**	Intervention adherence and tolerability (How was this assessed?)	line 78
	**10d**	Adverse and unanticipated events	line 78
**Discussion**	**11a**	A scientific discussion of the strengths AND limitations associated with this case report	linre 168-180
	**11b**	Discussion of the relevant medical literature **with references**.	line 108-124
	**11c**	The scientific rationale for any conclusions (including assessment of possible causes)	lines 91->107 112->124
	**11d**	The primary "take-away" lessons of this case report (without references) in a one paragraph conclusion	line 136-140
**Patient Perspective**	**12**	The patient should share their perspective in one to two paragraphs on the treatment(s) they received	line 144-145
**Informed Consent**	**13**	Did the patient give informed consent? Please provide if requested	**Yes** ☑ **No** ☐
